# Metastatic liver disease from non-colorectal, non-neuroendocrine, non-sarcoma cancers: a systematic review

**DOI:** 10.1186/s12957-015-0606-6

**Published:** 2015-05-29

**Authors:** Fabio Uggeri, Paolo Alessandro Ronchi, Paolo Goffredo, Mattia Garancini, Luca Degrate, Luca Nespoli, Luca Gianotti, Fabrizio Romano

**Affiliations:** Department of Surgery and Translational Medicine, University of Milano-Bicocca, San Gerardo Hospital, via Pergolesi 33, Monza, 20900 Italy; University of Milano-Bicocca, via Cadore 48, Monza, 20900 Italy; Department of Surgery, Duke University, Durham, USA; Department of Surgery, San Gerardo Hospital, via Pergolesi 33, Monza, 20900 Italy

**Keywords:** Liver metastases, Non-colorectal, Non-neuroendocrine, Non-sarcoma, Liver resection, Prognostic factors

## Abstract

**Background:**

Hepatic resection of liver metastases of non-colorectal, non-neuroendocrine, and non-sarcoma (NCNNNS) primary malignancies seems to improve survival in selected patients. The aims of the current review were to describe long-term results of surgery and to evaluate prognostic factors for survival in patients who underwent resection of NCNNNS liver metastases.

**Methods:**

We identified 30 full texts (25 single-center and 5 multicenter studies) published after year 1995 and published in English with a total of 3849 patients. For NCNNNS liver metastases, 83.4 % of these subjects were resected.

**Results:**

No prior systematic reviews or meta-analyses on this topic were identified. All studies were case series without matching control groups. The most common primary sites were breast (23.8 %), genito-urinary (21.8 %), and gastrointestinal tract (19.8 %). The median 5- and 10-year overall survival were 32.3 % (range 19–42 %) and 24 % (indicated only in two studies, range 23–25 %), respectively, with 71 % of R0 resections.

**Conclusions:**

There is evidence suggesting that surgery of NCNNNS metastases is safe, feasible, and effective if treatment is part of a multidisciplinary approach and if indication is based on the prognostic factors underlined in literature analysis.

## Review

Due to its filter role in the portal circulation, the liver is often the first organ involved in metastatic dissemination of gastrointestinal neoplasms. Hepatic resection is an established and recognized procedure for the treatment of colorectal liver metastases, and it is associated with higher survival rates than more conservative therapies. In fact, patients who undergo complete resections (R0) have a 5-year survival rate of approximately 40 % and a 10-year overall survival rate of 25 % [1–10]. The role of surgery for metastases from neuroendocrine neoplasms on long-term outcome is also well-documented [11, 12]. Recently, some authors analyzing liver metastases from sarcomas reported similar results to colorectal and neuroendocrine cancer with a 5-year survival ranging from 26 to 36 % [13, 14]. The increasing attention has led to a better standard of care. A multidisciplinary approach, surgical techniques, perioperative management, and technological advances have all contributed to the improvement of long-term survival after liver resections over the last two decades.

On the other hand, several other primary sites develop metastases in hepatic parenchyma. With regard to this third group of cancers (non-colorectal, non-neuroendocrine, and non-sarcomas (NCNNNS)), there is a paucity of data in medical literature. In fact, studies either had small number of patients likely due to the relative rarity and the lack of centralization in high volume centers, or they investigated diseases from primary tumors with different prognoses, including metastasis from cancer of the colorectum, neuroendocrine tissues, and sarcoma.

The aims of the current review were to describe long-term results of surgery and identify prognostic factors for survival in a heterogeneous group of liver metastases from NCNNNS primaries malignancies.

## Material and methods

### Literature search strategy

The original published studies were searched via PubMed and Medline databases, between 1995 and 2014. The following keywords were utilized: “liver” and “metastases”, “non-colorectal”, “non-neuroendocrine”, “non-sarcoma”, “hepatectomy” (or “resection”), and “prognostic factors”. More than 5000 references were identified. The reference lists of all retrieved articles were reviewed to further identify potentially relevant studies.

The purpose of data extraction has been to identify those items in which the percentage of patients who were resected for NCNNNS liver metastases was higher than 50 %.

### Selection criteria

Observational clinical studies that used hepatic resection as a therapeutic option for NCNNNS malignancies were identified for inclusion. All relevant prospective and retrospective series were also included. Specific inclusion criteria were studies published after the year 1995, human articles, and papers published in the English language.

Abstracts, reviews, letters, editorials, case reports, expert opinions, and articles contain short reviews were excluded. The final result is the analysis of 30 full texts: 25 single-center studies and 5 multicenter studies [15–44].

### Data extraction

Two reviewers independently appraised each article using similar protocols. Data extracted were: methodology, patient number and characteristics, outcomes, length of follow-up, overall survival or progression-free survival, mortality, morbidity, and prognostic factors. Median values and percentages were determined after tabulation of the results from the included studies. All the studies included in the present review aimed to demonstrate the efficacy of hepatic resection for liver metastases, although a minority evaluated also other concomitant ablative techniques.

## Results

**Fig. 1:**
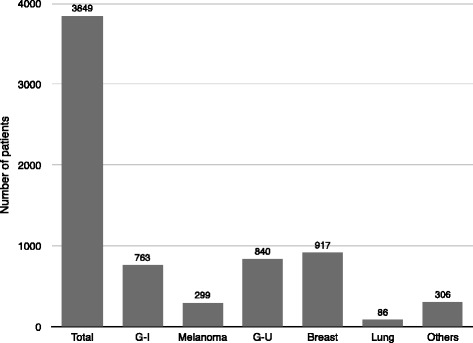
Total number of patients and origins of metastases

The 30 studies included a total of 3849 patients (Fig. 1). All studies were case series without matching control groups: 11 studies described more than 100 patients [16, 24, 26, 29, 34–37, 41, 43, 44], 6 studies between 50 and 100 patients [15, 17, 31, 38, 39, 42], and 13 studies less than 50 patients [18–23, 25, 27, 28, 30, 32, 33, 40]. No prior systematic reviews or meta-analyses on this topic were identified. The largest series was published by Adam et al. in 2006 [37] including 1452 patients who underwent surgical procedures. In 22 studies, patients candidates to hepatic resection of liver metastases (LM) were the target population, but subjects undergoing alternative, additional surgical procedures (mainly radiofrequency ablation or cryoablation in addition to partial hepatectomy) or palliative treatment was included for the purpose of comparison [15, 20–31, 34–37, 39–43]. Study objectives and inclusion criteria were clearly described in all the studies.

Twenty-eight studies reported overall survival results (Table 1), which were unclear in one article [23]. Twenty-one studies reported data on progression-free survival [16–20, 24, 25, 29, 31–33, 35–44]. Each article reported length of follow-up of at least 5 years. The 5- and 10-year overall survival ranges were 19–42 % and 23–25 %, respectively, with a mean 5-year overall survival of 32.3 % (Table 1).

**Table 1:** Overall and disease-free survival

First author	Overall survival						Disease-free survival					
	Median (months)	1 year (%)	2 years (%)	3 years (%)	5 years (%)	10 years (%)	Median (months)	1 year (%)	2 years (%)	3 years (%)	5 years (%)	10 years (%)
Harrison [38]	32	80	–	45	37	–	–	–	–	–	–	18.7
Lindell [27]	32	75	–	–	36	25	–	–	–	–	–	–
Elias [24]	–	–	–	–	36	–	–	–	–	–	28	–
Berney [19]	19	61	43	–	27	–	36^a^	–	–	–	–	–
Hamy [22]	19.6	54 ± 8	42 ± 8	–	27 ± 8	–	–	–	–	–	–	–
Benevento [25]	38 ± 11	54	42	–	21	–	–	–	–	–	–	–
Hemming [40]	46	85	–	55	45	–	28^a^	–	–	–	–	–
Takada [23]	0–53	–	–	–	–	–	–	–	–	–	–	–
Van Ruth [18]	21	–	–	–	35	–	12	–	–	–	20	–
Laurent [30]	–	81	–	40	35	–	–	–	–	–	–	–
Goering [20]	45	82	–	55	39	–	–	43	–	21	–	–
Karavias [28]	–	–	–	78	–	–	–	–	–	–	–	–
Torras [33]	–	86	48	–	–	–	53 ± 38	50	25	–	–	–
Yedibela [16]	23	–	49	–	26	–	25	–	–	–	–	–
Weitz [35]	42	–	–	57	–	–	17	–	–	30	–	–
Cordera [31]	28.8	81.1	–	43	30.2	–	13.2	64.7	–	–	15.8	–
Earle [17]	36	88.5	–	49.1	34.9	–	21.5	–	–	–	–	–
Adam [37]	35	–	–	–	36	23	13	–	–	–	21	15
Verhoef [21]	37	–	–	–	42	–	–	–	–	–	–	–
Lendoire [34]	27	67	–	34	19	–	–	–	–	–	–	–
O’Rourke [29]	42	–	–	56.1	38.5	–	18	–	–	37.2	26.5	–
Pais Costa [32]	–	–	–	50	–	–	–	–	–	40	–	–
Ercolani [41]	35.5 ± 6.4	83.6	–	56.5	40	–	26.6 ± 4.1	75	–	44	30	–
Duan [26]	38.8 ± 26.7	84.8	–	44.7	29.5	–	–	–	–	–	–	–
Bresadola [39]	20	71.9	–	42.8	28.9	–	19–44	68–85	–	29–63	19–52	–
Marudanayagam [42]	19	72.9	–	47.9	25.6	–	19	–	–	–	–	–
Treska [15]	–	88.6	–	72.5	36.9	–	–	–	–	–	–	–
Groeschl [36]	49	73	–	50	31	–	23	–	–	–	–	–
Slotta [43]	20.5	66	–	43	30	–	–	–	–	–	39	–
Takemura [44]	41.8	83.9	–	55.4	41	–	10	43.7	–	21.1	18.1	–
Range	0–53				19–42	23–25	10–53				15.8–52	15–18.7
Average	32.3				32.3	24	23.1				26	16.9

^a^Only metachronous group

**Fig. 2:**
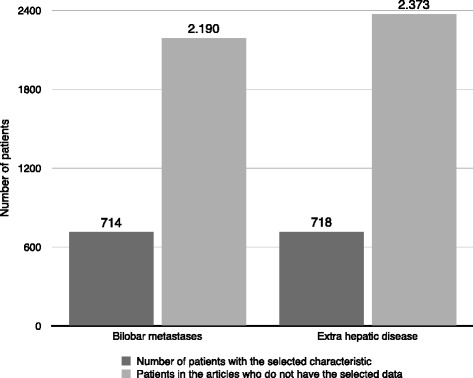
Metastatic involvement

**Fig. 3:**
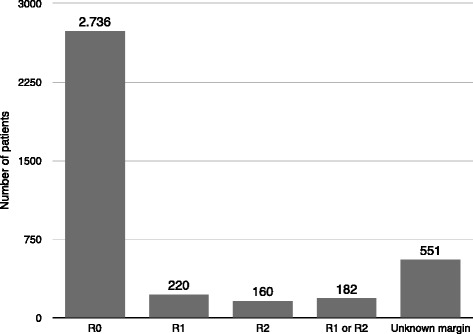
Resection margin

Figures 1 and 2 present the results of the primary sites and systemic spread of metastases. The most common primary sites were breast (23.8 %), genito-urinary (21.8 %), and gastrointestinal tract (19.8 %). Sixteen studies included patients with extrahepatic metastases [16–18, 24, 25, 27–31, 33, 36, 37, 42–44], whereas fourteen studies included patients with isolated liver disease only, or the presence of extrahepatic metastases at the time of hepatic resection was not specified [15, 19–23, 25, 26, 32, 34, 38–41]. The presence of extrahepatic disease wildly ranged between 0 and 53.1 % because several authors considered it an exclusion criterion for surgery, while others did not deem extrahepatic disease a contraindication or a negative prognostic factor. Therefore, liver resection was performed with the intent to achieve a radical resection (R0). Metastases affected two hepatic lobes in 0–37.5 % of patients. The presence of bilobar metastases or extrahepatic disease may not be considered as a contraindication for surgery [16, 17, 29–31, 33, 34, 38, 40, 42, 44]; 18 % of authors resected bilobar diseases, otherwise considering this criteria a prognostic factor that impact prognosis. Figure 3 also shows the results regarding the status of the surgical margin: although an R0 resection is always the primary objective of the surgeon, it is obtained in 2736 of the 3849 patients, although it is specified only in 21 studies.

A detailed evaluation of significant and non-significant prognostic factors for overall survival and progression-free survival is presented in Table 2. By the univariate analysis, the most common factors (reported in ≥3 studies) associated with poor survival analysis were the following: age, synchronous metastatic disease, site of the primary tumor, presence of symptoms, type and extension of hepatectomy, macroscopic (R2) residual disease and distance of free-surgical margin, adjuvant treatment, presence of extrahepatic disease, number of hepatic lesions and the size of the greatest one, and bilobar disease.

**Table 2:** Prognostic factors

Prognostic factor	Variables that influence overall survival	Variables that influence progression-free survival
Sex	39	35
Preoperative treatment of liver	15	
Metastatic situation: synchronous/metachronous	17, 23, 27, 34, 38	44
Primary site and histological subtype	15–26, 33, 34, 37–41, 43	31, 35
Symptomatic at the time of resection	41^a^	
Hepatic involvement	25, 26	
Type of the intervention performed	17, 37, 39–41	
Macroscopically incomplete resection (R2)	17, 19, 38, 40	29
Surgical margin status	16, 17, 19, 32, 34, 37, 38, 40, 42	35
Adjuvant treatment	15, 17, 18, 20, 21, 24–26, 37	44
Presence of extrahepatic disease	15, 16, 20, 27, 28, 30, 37	
Surgery timing	19, 22, 26, 31	31
Postoperative complications	17, 44	44
Number of metastases	17, 19, 26, 27, 33, 39, 44	44
Size of metastases	26, 29, 36, 41, 42	29, 44
Presence of vascular invasion	36	
Lymph node metastases	29, 36, 39	29
Disease-free survival	26, 30, 37, 38, 41	17, 31, 35, 44
Blood transfusion	44	44

^a^Symptoms within 1 year

In particular, we analyzed the five main prognostic factors as follows:

- primary site and histological subtype (22 articles, Table 2); liver metastases from breast kidney, uterus, ovary, testicle, ampulla of Vater, and adrenal gland cancer had a 5-year overall survival greater than 30 %, while metastases from gastric and duodenum cancer had a survival between 30 and 15 %; liver metastases from pancreas, anus, esophagus, cardia, and lung cancers had a 5-year overall survival less than 15 % [37, 43].
- size and number of metastases (11 articles, Table 2); the prognosis worsened especially when the number of metastases was higher than 3–4 and the size greater than 5–6 cm.
- surgical margin status (10 articles, Table 2); to perform surgery with curative intent, it is necessary to accomplish disease-free resection margins (R0). Nevertheless, R0 resection was reported with a range between 55.9 [19] and 100 % of cases [28, 30, 32], showing that a complete excision of the lesion is not always achievable suggesting that the margin of resection could be an important prognostic factor, on overall survival.
- type of the intervention performed (5 articles, Table 2); the extent of hepatectomy was also an independent negative prognostic factor, possibly reflecting the magnitude of tumor burden [37].
- time of metastasis appearance (6 articles, Table 2). Despite the presence of synchronous metastases might represent the disease aggressiveness, the 5-year overall survival in patients with metachronous disease or synchronous disease were 31–37 % and 31–36 %, respectively [16, 37].

## Discussion

The role of surgery in the management of liver metastases from colorectum (CR) or neuroendocrine tumors has been well described in the literature; several studies have focused their attention on the surgical indications of these patients and the respective outcomes, showing the need for an aggressive surgical therapy. In particular, surgical treatment of metastatic CR cancer has improved long-term outcome. A 5-year survival rate was 25 % in the 80s, progressively increased up to 47 % in 2008 [7, 45–51]. In contrast, the treatment of NCNNNS liver metastases does not have a clearly defined role, mostly because of discrepant characteristics of patients, difficulty in their selection, and lack of high volume series.

The aim of this review is to help shedding light on this controversial topic not adequately outlined so far.

Several multicenter studies and reviews report a survival rate ranging from 27.9 to 49.3 % in patients with metastases from non-CR cancer, depending on the tumor histology [5]. Although the set of “non-CR metastases” is extremely heterogeneous, there is a general agreement on the surgical treatment of some homogeneous groups of tumors such as metastases from neuroendocrine tumors and sarcoma. In these cases, the resection has been proved safe and able to prolong survival compared to non-invasive treatments, obtaining an overall survival rate at 5-year of 20–33 % and of 46–86 % for sarcoma and neuroendocrine tissues, respectively [11–14, 24, 52].

The results of different studies suggest that the technological and cultural evolutions in surgery has improved the prognosis in patients treated from 1978 to 2014, especially in terms of 5-year overall survival. The most recent data on the treatment of metastatic non-CR cancer are comparable to those reported in the literature in studies on the treatment of metastatic CR cancer dated 15–20 years ago [53–55].

Beyond the heterogeneity in terms of histology of the primary disease and the stage of cancer, the improvement in the survival rates of patients undergoing surgery strengthens and demonstrates the utility of resection with a 19–42 % 5-year survival rates.

The theory defining tumor spreading to the liver as a “systemic” and not a “regional” disease is obsolete and justifies why liver metastases were not surgically removed until some decades ago. Despite the presence of metastases representing an advance disease and that hematogenous dissemination may be a contraindication for surgery, the most recent results suggest that surgery, combined by chemotherapy, is useful in this type of systemic diseases by improving overall.

The treatment of liver non-CR metastases remained a debated topic until about 15 years ago, when improved survival was reported among patients operated on for hepatic metastases from testicular, kidney, and breast cancers [19, 24].

The surgical approach to non-CR liver metastases is essentially based on two fundamental issues that have become increasingly important in the last two decades:

- Hepatic resection has become safer thanks to improved surgical techniques and an accurate pre-and intraoperative imaging allowing parenchyma spearing.
- The complementary role of new and effective chemotherapy agents and surgery for some non-CR tumors

Therefore, it appears that the role of surgery in the treatment of liver metastases may be a relevant hope for patients when they meet the criteria of resectability, regardless the number of metastases; further efforts should be pursued to better evaluate its clinical impact and standardize management protocols.

Regarding surgical margin, it is considered safe when >0.5–1 cm since shorter margins are associated with higher rates of recurrence. However, as already shown for colorectal cancer, the overall survival rate seems to be not significantly influenced by the margin width but more by the presence of residual disease (R1-R2 versus R0) [17, 19, 34, 37, 38, 40, 42]. As such, it might be that 1 mm is sufficient to improve survival. In addition, the more patients with advanced diseases (multiple metastases, bilobar, or large) are resected, the more clearly liver parenchyma must be preserved, obtaining less wide margins. Debulking surgery is a fairly debated topic, because up to date, it has not been demonstrated to impact survival results [14, 56, 57], unless an R0 resection is unachievable with adjuvant/neoadjuvant [13] and medical therapy has failed.

Contraindications to debulking surgery seem to apply in particular to rapidly progressive metastatic disease not controlled by systemic treatments and to synchronous liver metastases, except for breast [37] and genito-urinary tract [43] neoplasms because the efficacy of medical treatments allow to improve survival and to stretch the limit of surgery.

Up-front hepatic resection seems contraindicated in patients with major comorbidities or for tumors with advanced invasion of major vessels and in those with an expected residual liver volume less than 30–40 % after resection. In the latest cases, neoadjuvant chemotherapy or other cytoreductive approaches may allow delayed reexamination and new judgment of operability and resectability. However, in some chemoresistant tumors (melanomas, kidney), surgery should be considered, when feasible, as the only possible therapy with curative intent and should not be delayed.

The presence of extrahepatic disease seems to be a relative contraindication to liver surgery.

An half of the studies analyzed deem the presence of an extrahepatic an absolute contraindication to surgical resection, while the remnant studies push the limit of surgical approach with the aim to obtain an R0 resection.

In our univariate analysis, the most common prognostic factors related to the 5-year overall survival rate was the site of the primitive tumor and the histological subtype. In particular, the worse prognosis was for hepatic metastases from gastrointestinal tumors, except for well-selected patients with metachronous metastases from gastric cancer.

The low rate of complication after elective liver surgery and the survival benefit observed in association with hepatectomy with more than one third of patients alive at 5 years and subsequently a quarter to 10 years support the inclusion of surgery in a multidisciplinary set of care for these patients.

## Conclusions

Liver resection for NCNNNS metastases seems to be safe and feasible. Long-term outcome is deeply affected by some clinical and pathological features, such as the histology of the primary tumor. Statistical models based on correct patient prognostic factors can help to predict long-term survival.

Currently, there is evidence that the surgery of NCNNNS metastases is effective if the indication is based on the evaluation of precise risk factors and if surgery is associated with complementary therapies. The major benefits are accomplished for genito-urinary and breast cancer, for size of mestastases less than 5 cm, a curative resection is achieved and when the appearance of hepatic lesions is longer than 12 months from primary tumors.
